# Supplementary material for: Frontoparietal connectivity as a product of convergent evolution in rodents and primates: functional connectivity topologies in grey squirrels, rats, and marmosets
Source: Commun Biol. 2022 Sep 17;5:986. doi: 10.1038/s42003-022-03949-x (PMC9482620; doi:10.1038/s42003-022-03949-x)
Supplement: Supplementary file 2 — Supplementary Information [file 42003_2022_3949_MOESM2_ESM.pdf]

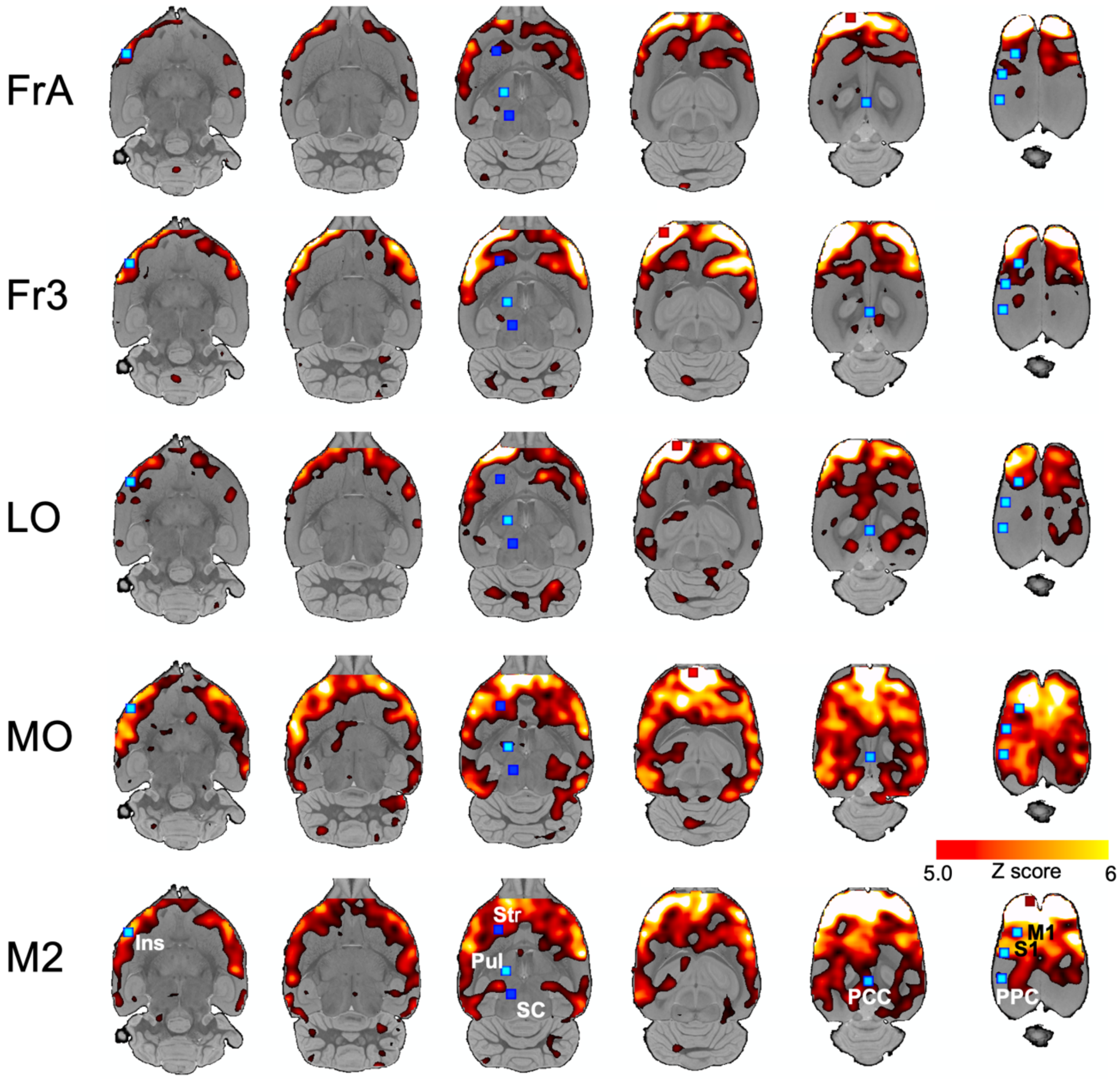

Supplementary Figure 1. Rat whole-brain functional connectivity of frontal seed areas. The mean time courses were extracted from each seed, then functional connectivity values were calculated with the rest of the brain. Functional connectivity (warm colors) overlaid on axial slices of the rat template brain. The bottom panel shows the location of each of the regions of interest used in the fingerprint analysis. Seed region abbreviations: Frontal association cortex (FrA); lateral

orbital cortex (LO); medial orbital cortex (MO); frontal cortex, area 3 (Fr3); secondary motor cortex (M2).

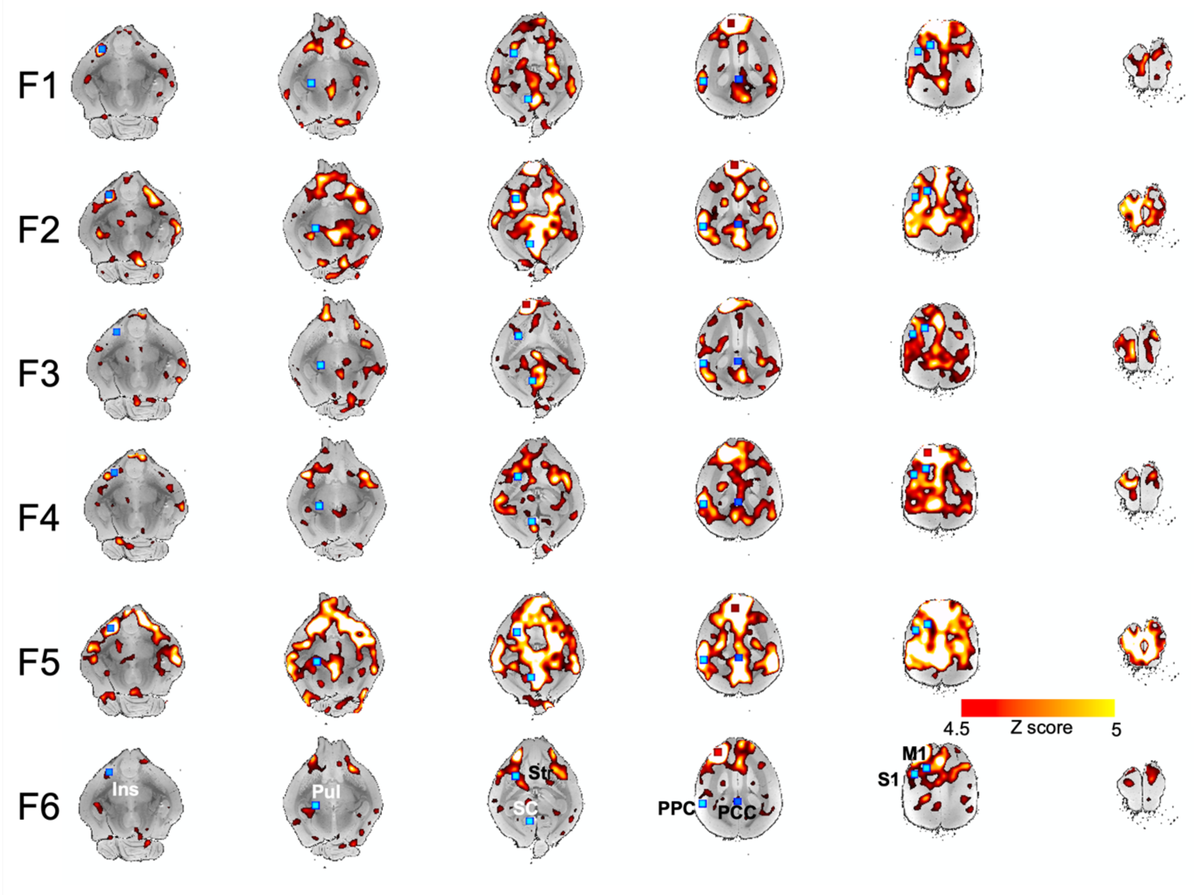

Supplementary Figure 2. Squirrel whole-brain functional connectivity of frontal seed regions. Mean time courses were extracted from each seed, then functional connectivity values were calculated with the rest of the brain. Functional connectivity (warm colors) overlaid on axial slices of the high-resolution ex-vivo squirrel brain. The bottom panel shows the location of each of the regions of interest used in the fingerprint analysis. Seed region abbreviations: frontal areas 1 – 6 (F1-F6).

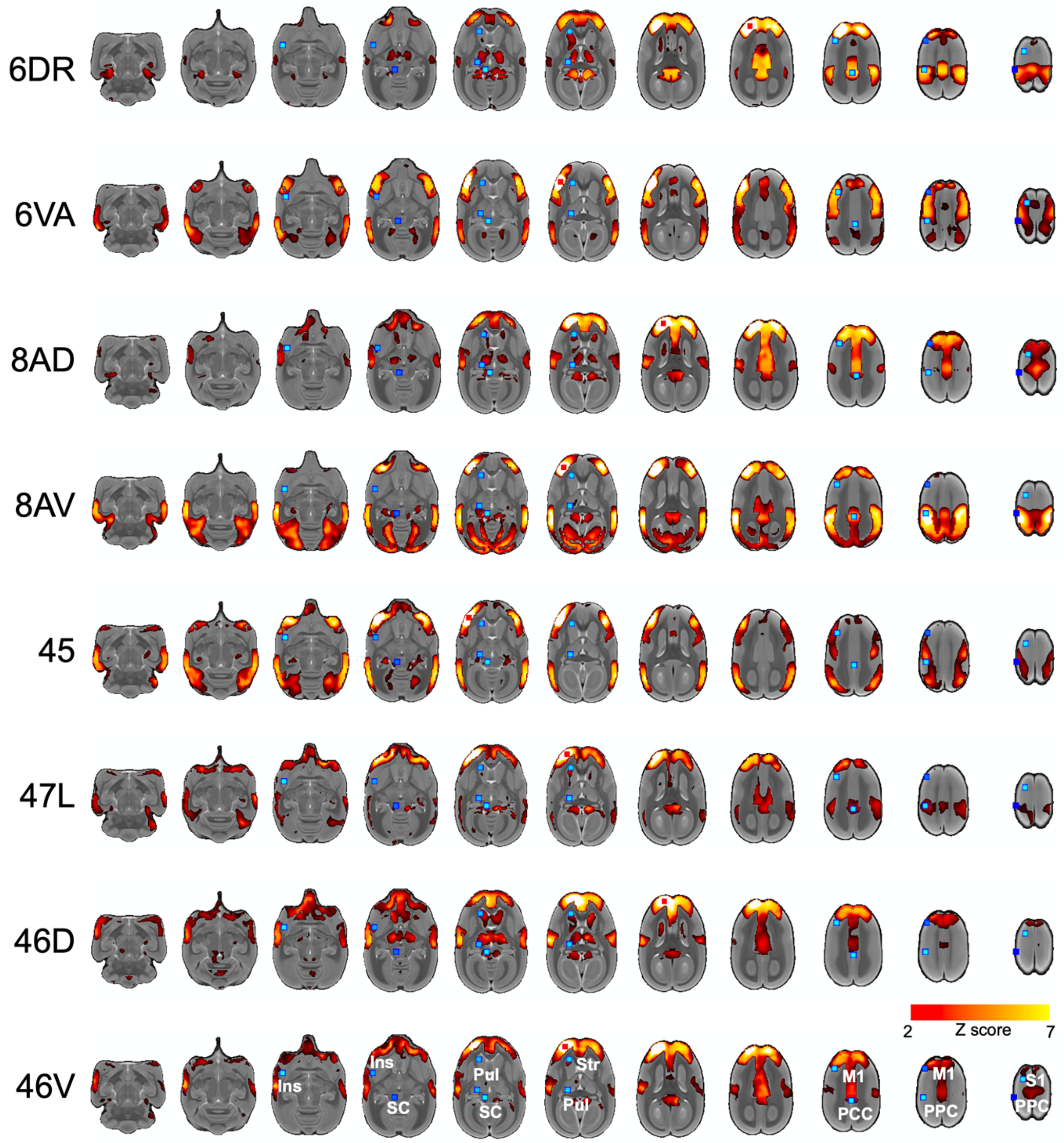

Supplementary Figure 3. Marmoset whole-brain functional connectivity of frontal seed areas. The mean time courses were extracted from each seed, then functional connectivity values were calculated with the rest of the brain. Functional connectivity (warm colors) overlaid on axial slices of the marmoset template brain. The bottom panel shows the location of each of the

regions of interest used in the fingerprint analysis. Seed region abbreviations: area 6 of cortex, ventral, part a (6VA); area 6 of cortex, dorsorostral part (6DR); area 8a or cortex, dorsal part (8AD); area 8a of cortex, ventral part (8AV); area 45 of cortex (45); area 47 of cortex (47); area 46 of cortex, dorsal part (46D); area 46 of cortex, ventral part (46V).

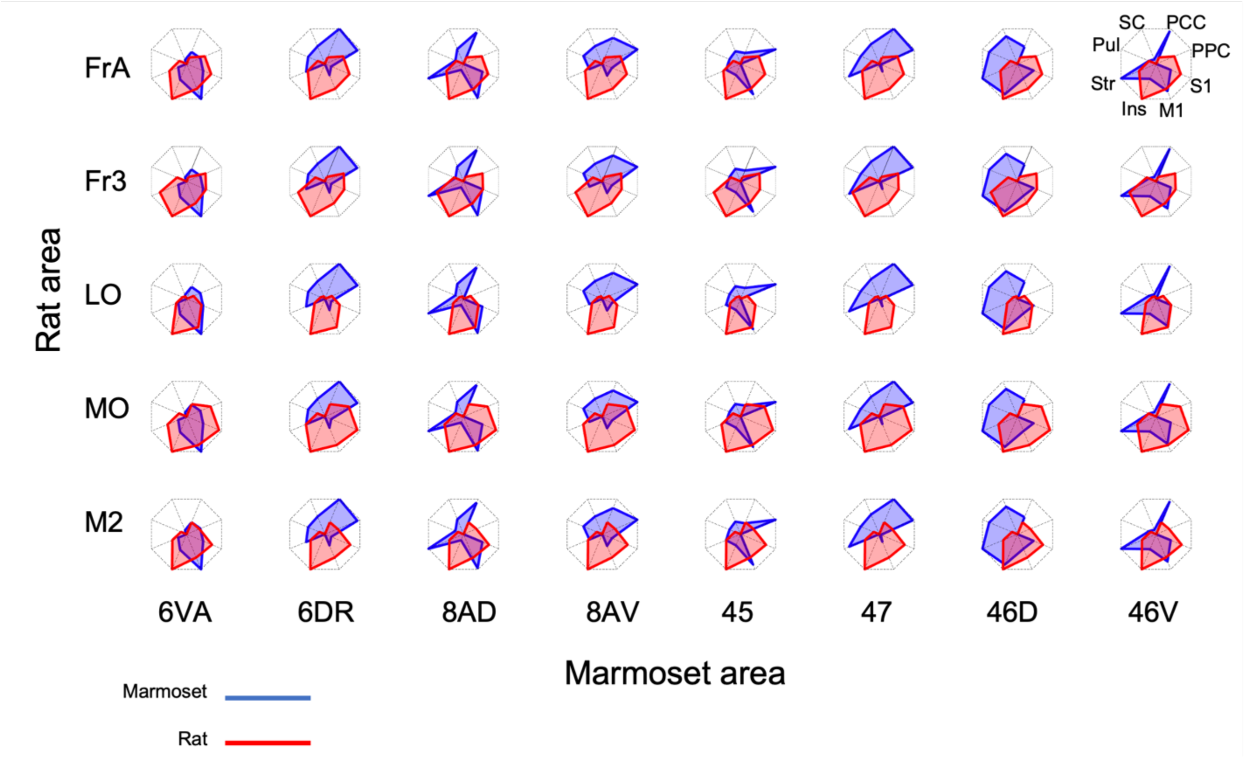

Supplementary Figure 4. Comparison of interareal functional connectivity patterns between rats and marmosets. Note that for each species, the fingerprints are normalized between 0 and 1 to allow for pattern comparability: A 0 value does not necessarily mean that there was no activation in a region, but just that the region had the lowest relative value within that fingerprint. Seed region abbreviations: Frontal association cortex (FrA); lateral orbital cortex (LO); medial orbital cortex (MO); frontal cortex, area 3 (Fr3); secondary motor cortex (M2); area 6 of cortex, ventral, part a (6VA); area 6 of cortex, dorsorostral part (6DR); area 8a or cortex, dorsal part (8AD); area 8a of cortex, ventral part (8AV); area 45 of cortex (45); area 47 of cortex (47); area 46 of cortex, dorsal part (46D); area 46 of cortex, ventral part (46V).

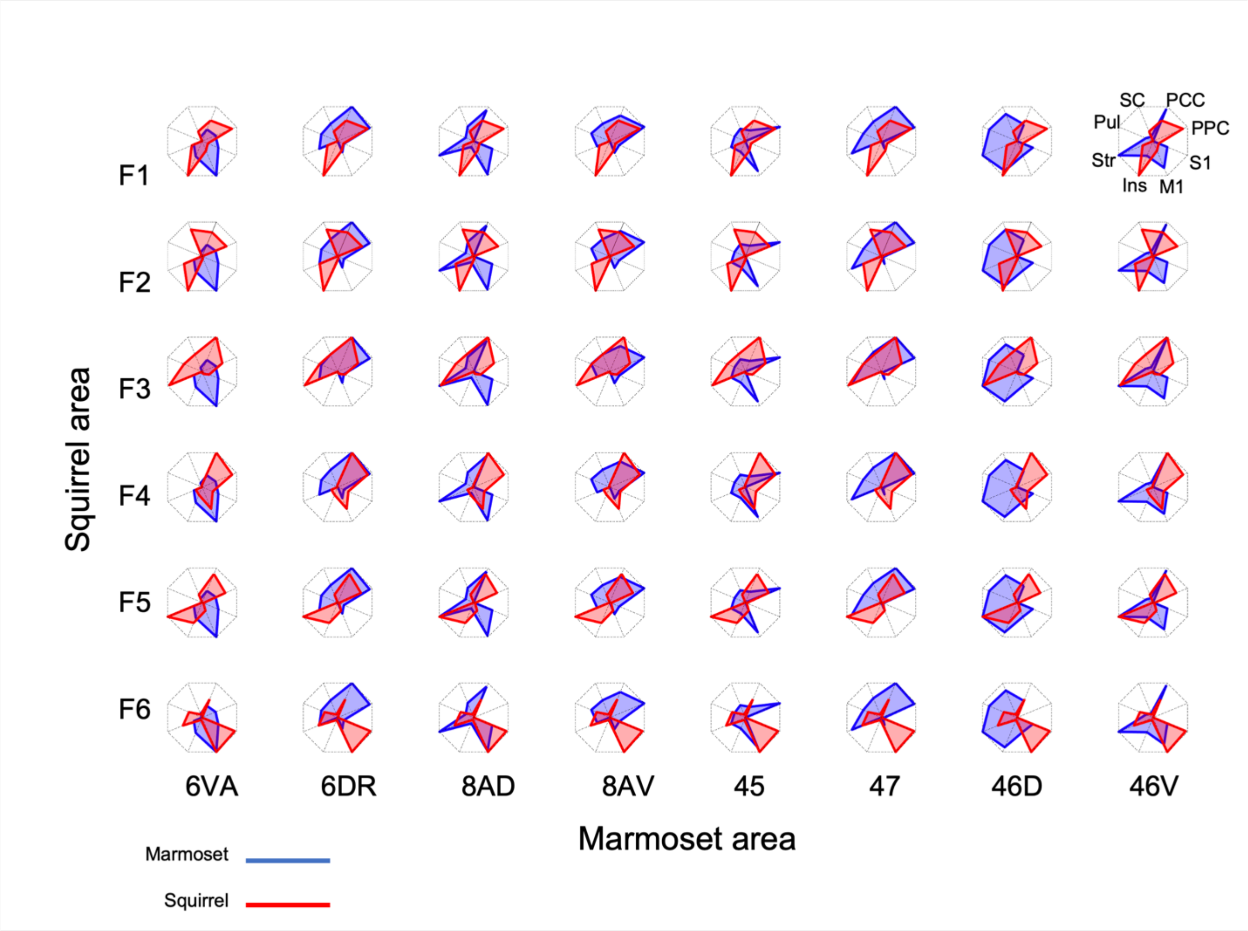

Supplementary Figure 5. Comparison of interareal functional connectivity patterns between squirrels and marmosets. Note that for each species, the fingerprints are normalized between 0 and 1 to allow for pattern comparability: A 0 value does not necessarily mean that there was no activation in a region, but just that the region had the lowest relative value within that fingerprint. Seed region abbreviations: frontal areas 1 – 6 (F1-F6); area 6 of cortex, ventral, part a (6VA); area 6 of cortex, dorsorostral part (6DR); area 8a or cortex, dorsal part (8AD); area 8a of cortex, ventral part (8AV); area 45 of cortex (45); area 47 of cortex (47); area 46 of cortex, dorsal part (46D); area 46 of cortex, ventral part (46V).

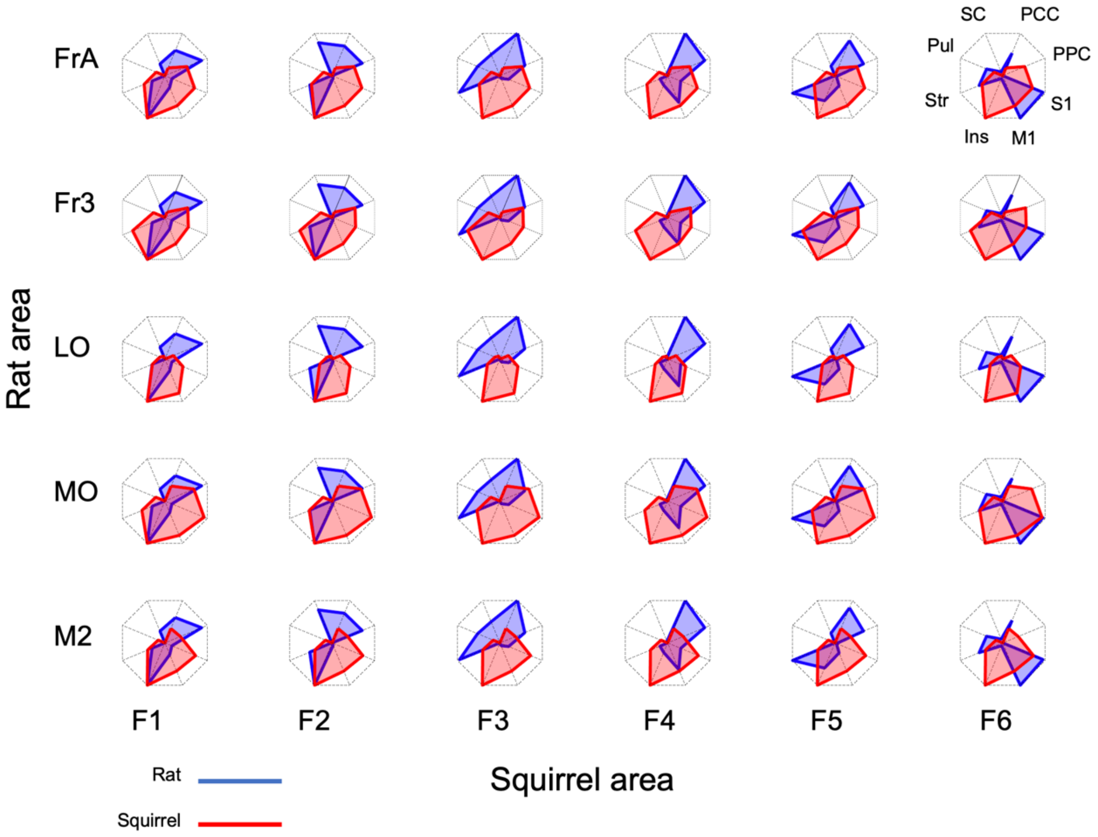

Supplementary Figure 6. Comparison of interareal functional connectivity patterns between rats and squirrels. Note that for each species, the fingerprints are normalized between 0 and 1 to allow for pattern comparability: A 0 value does not necessarily mean that there was no activation in a region, but just that the region had the lowest relative value within that fingerprint. Seed region abbreviations: Frontal association cortex (FrA); lateral orbital cortex (LO); medial orbital cortex (MO); frontal cortex, area 3 (Fr3); secondary motor cortex (M2); frontal areas 1 – 6 (F1-F6).

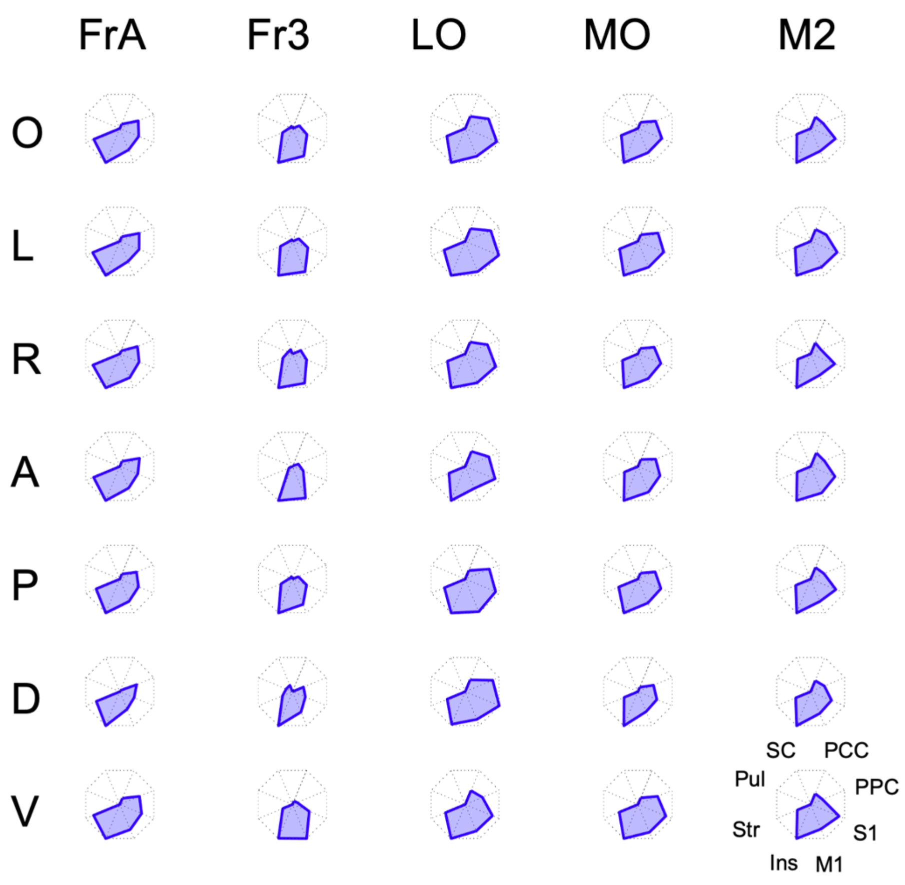

Supplementary Figure 7. Resulting change in fingerprint by translating the seed (columns) by one voxel in rats. O = original; L = left; R = right; A = anterior; P = posterior; D = dorsal; V = ventral. Seed region abbreviations: Frontal association cortex (FrA); lateral orbital cortex (LO); medial orbital cortex (MO); frontal cortex, area 3 (Fr3); secondary motor cortex (M2).

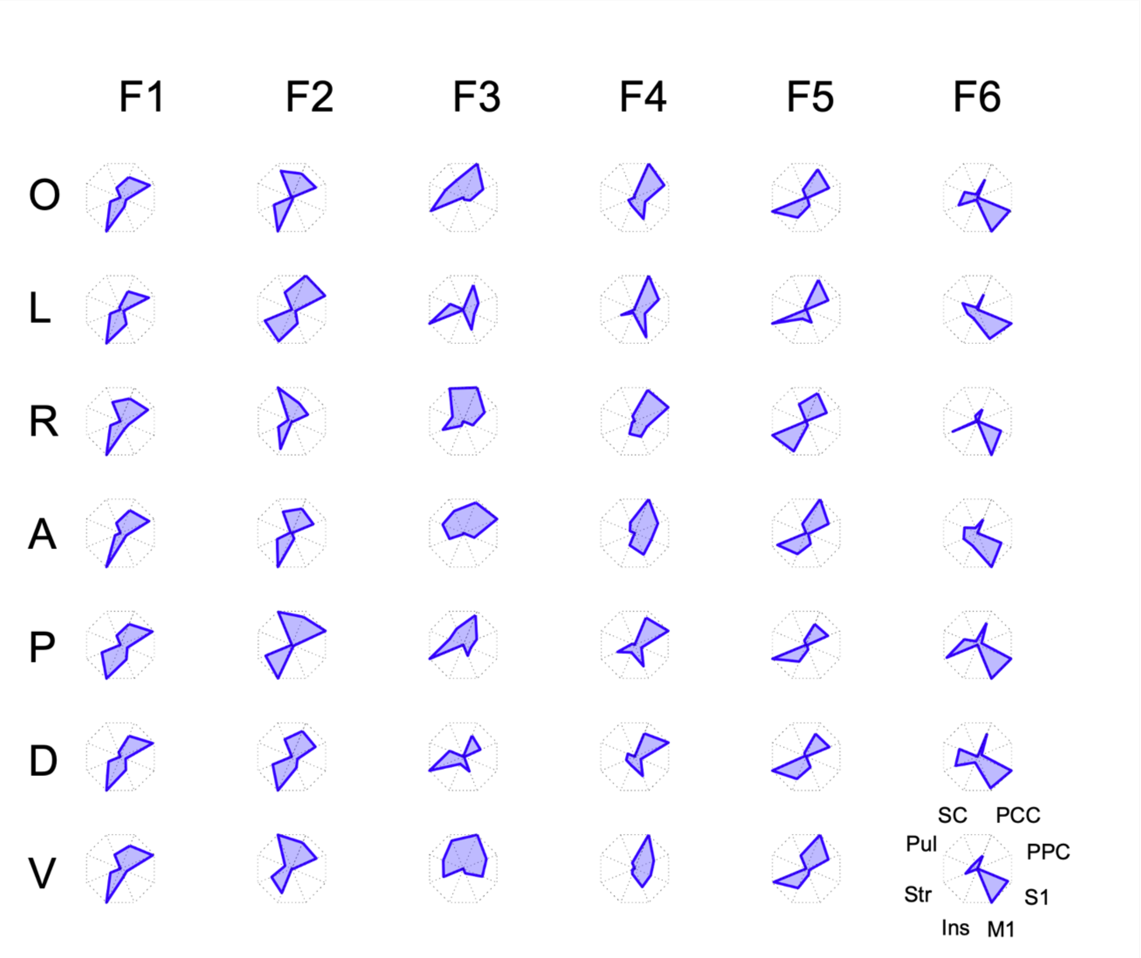

Supplementary Figure 8. Resulting change in fingerprint by translating the seed (columns) by one voxel in squirrels. O = original; L = left; R = right; A = anterior; P = posterior; D = dorsal; V = ventral. Seed region abbreviations: frontal areas 1 – 6 (F1-F6).

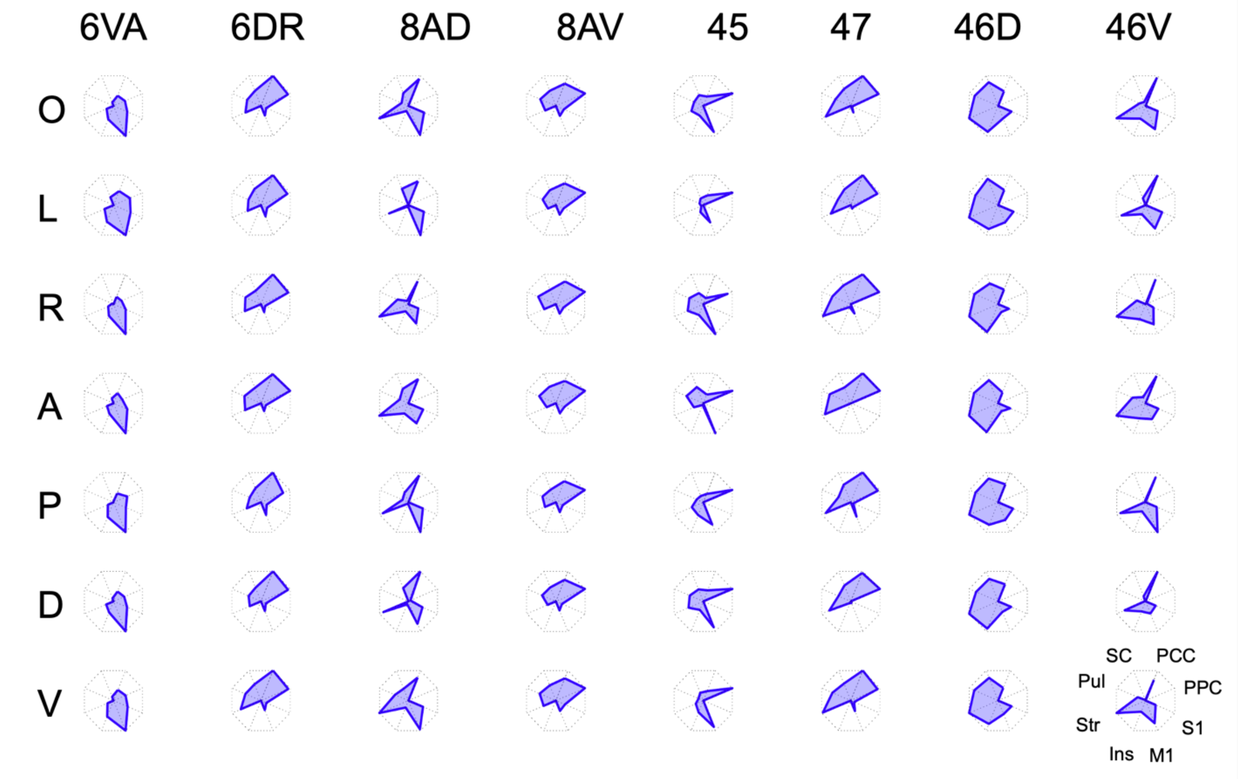

Supplementary Figure 9. Resulting change in fingerprint by translating the seed (columns) by one voxel in marmosets. O = original; L = left; R = right; A = anterior; P = posterior; D = dorsal; V = ventral. Seed region abbreviations: area 6 of cortex, ventral, part a (6VA); area 6 of cortex, dorsorostral part (6DR); area 8a or cortex, dorsal part (8AD); area 8a of cortex, ventral part (8AV); area 45 of cortex (45); area 47 of cortex (47); area 46 of cortex, dorsal part (46D); area 46 of cortex, ventral part (46V).

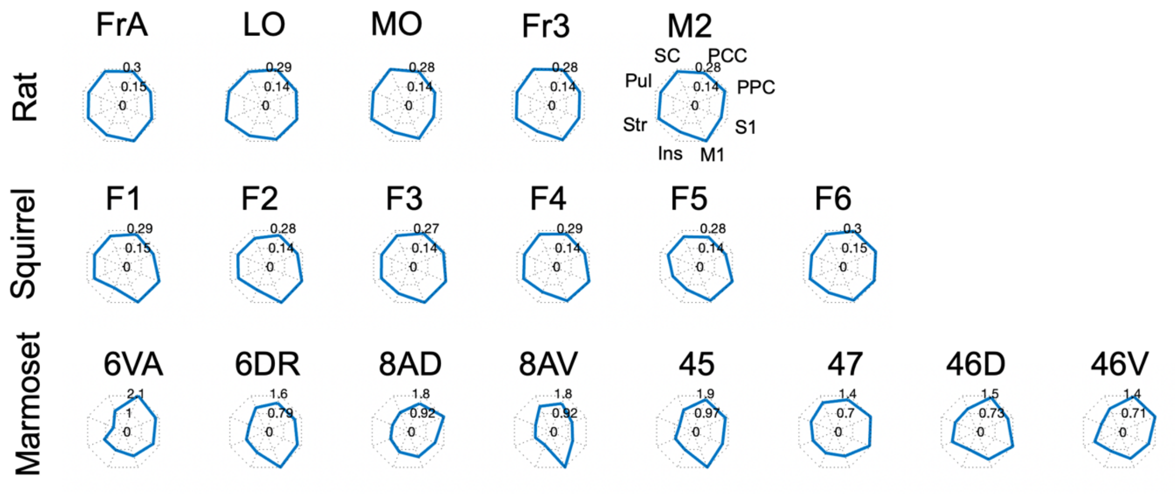

Supplementary Figure 10. Variance plotted for each fingerprint, in each species. Seed region abbreviations: Frontal association cortex (FrA); lateral orbital cortex (LO); medial orbital cortex (MO); frontal cortex, area 3 (Fr3); secondary motor cortex (M2); frontal areas 1 – 6 (F1-F6); area 6 of cortex, ventral, part a (6VA); area 6 of cortex, dorsorostral part (6DR); area 8a or cortex, dorsal part (8AD); area 8a of cortex, ventral part (8AV); area 45 of cortex (45); area 47 of cortex (47); area 46 of cortex, dorsal part (46D); area 46 of cortex, ventral part (46V).
