# Supplementary material for: Frontoparietal connectivity as a product of convergent evolution in rodents and primates: functional connectivity topologies in grey squirrels, rats, and marmosets
Source: Commun Biol. 2022 Sep 17;5:986. doi: 10.1038/s42003-022-03949-x (PMC9482620; doi:10.1038/s42003-022-03949-x)
Supplement: Supplementary file 3 — Reporting Summary [file 42003_2022_3949_MOESM3_ESM.pdf]

nature portfolio

Corresponding author(s): David Schaeffer

Last updated by author(s): Jul 18, 2022

Reporting Summary

Nature Portfolio wishes to improve the reproducibility of the work that we publish. This form provides structure for consistency and transparency in reporting. For further information on Nature Portfolio policies, see our Editorial Policies and the Editorial Policy Checklist.

Statistics

For all statistical analyses, confirm that the following items are present in the figure legend, table legend, main text, or Methods section.

n/a	Confirmed	
☐	☒	The exact sample size (n) for each experimental group/condition, given as a discrete number and unit of measurement
☐	☒	A statement on whether measurements were taken from distinct samples or whether the same sample was measured repeatedly
☐	☒	The statistical test(s) used AND whether they are one- or two-sided<br>*Only common tests should be described solely by name; describe more complex techniques in the Methods section.*
☐	☒	A description of all covariates tested
☒	☐	A description of any assumptions or corrections, such as tests of normality and adjustment for multiple comparisons
☐	☒	A full description of the statistical parameters including central tendency (e.g. means) or other basic estimates (e.g. regression coefficient) AND variation (e.g. standard deviation) or associated estimates of uncertainty (e.g. confidence intervals)
☒	☐	For null hypothesis testing, the test statistic (e.g. F, t, r) with confidence intervals, effect sizes, degrees of freedom and P value noted<br>*Give P values as exact values whenever suitable.*
☒	☐	For Bayesian analysis, information on the choice of priors and Markov chain Monte Carlo settings
☒	☐	For hierarchical and complex designs, identification of the appropriate level for tests and full reporting of outcomes
☒	☐	Estimates of effect sizes (e.g. Cohen's d, Pearson's r), indicating how they were calculated

Our web collection on statistics for biologists contains articles on many of the points above.

Software and code

Policy information about availability of computer code

Data collection	Paravision Version 6.
Data analysis	FMRIB Software Library v6.0<br>AFNI Version AFNI_20.0.19 'Galba'<br>ANTs Version 5<br>Matlab 2019b<br>Python 2.7.16

For manuscripts utilizing custom algorithms or software that are central to the research but not yet described in published literature, software must be made available to editors and reviewers. We strongly encourage code deposition in a community repository (e.g. GitHub). See the Nature Portfolio guidelines for submitting code & software for further information.

Data

Policy information about availability of data

All manuscripts must include a data availability statement. This statement should provide the following information, where applicable:

- Accession codes, unique identifiers, or web links for publicly available datasets
- A description of any restrictions on data availability
- For clinical datasets or third party data, please ensure that the statement adheres to our policy

Data available on request from the authors.

Human research participants

Policy information about studies involving human research participants and Sex and Gender in Research.

Reporting on sex and gender	*Use the terms sex (biological attribute) and gender (shaped by social and cultural circumstances) carefully in order to avoid confusing both terms. Indicate if findings apply to only one sex or gender; describe whether sex and gender were considered in study design whether sex and/or gender was determined based on self-reporting or assigned and methods used. Provide in the source data disaggregated sex and gender data where this information has been collected, and consent has been obtained for sharing of individual-level data; provide overall numbers in this Reporting Summary. Please state if this information has not been collected. Report sex- and gender-based analyses where performed, justify reasons for lack of sex- and gender-based analysis.*
Population characteristics	*Describe the covariate-relevant population characteristics of the human research participants (e.g. age, genotypic information, past and current diagnosis and treatment categories). If you filled out the behavioural & social sciences study design questions and have nothing to add here, write "See above."*
Recruitment	*Describe how participants were recruited. Outline any potential self-selection bias or other biases that may be present and how these are likely to impact results.*
Ethics oversight	*Identify the organization(s) that approved the study protocol.*

Note that full information on the approval of the study protocol must also be provided in the manuscript.

Field-specific reporting

Please select the one below that is the best fit for your research. If you are not sure, read the appropriate sections before making your selection.

☒ Life sciences ☐ Behavioural & social sciences ☐ Ecological, evolutionary & environmental sciences

For a reference copy of the document with all sections, see nature.com/documents/nr-reporting-summary-flat.pdf

Life sciences study design

All studies must disclose on these points even when the disclosure is negative.

Sample size	Sample size was determined by repeatability of spatial fMRI patterns at the individual animal level.
Data exclusions	All acquired data were included.
Replication	Group results were repeatable at the individual animal level
Randomization	Selection not randomized; available animals used.
Blinding	Experimenters were not blinded during the experiment, but identifiers were randomly assigned for analysis

Reporting for specific materials, systems and methods

We require information from authors about some types of materials, experimental systems and methods used in many studies. Here, indicate whether each material, system or method listed is relevant to your study. If you are not sure if a list item applies to your research, read the appropriate section before selecting a response.

Materials & experimental systems

n/a	Involved in the study	
☒	☐	Antibodies
☒	☐	Eukaryotic cell lines
☒	☐	Palaeontology and archaeology
☐	☒	Animals and other organisms
☒	☐	Clinical data
☒	☐	Dual use research of concern

Methods

n/a	Involved in the study	
☒	☐	ChIP-seq
☒	☐	Flow cytometry
☐	☒	MRI-based neuroimaging

Animals and other research organisms

Policy information about studies involving animals; ARRIVE guidelines recommended for reporting animal research, and Sex and Gender in Research

Laboratory animals	Data were collected from five adult male Wistar rats aged 8-12 weeks and weighing from 250-350 g. Data were collected from 31 adult marmosets, 8 females, aged 14 -115 months, weighing from 240 -625 g.
Wild animals	Data were collected from five (1 female, 4 melanistic) adult wild-caught Eastern grey squirrels (Sciurus carolinensis) of unknown age weighing 590 – 700 g under a wildlife scientific collector's authorization from the Ontario Ministry of Natural Resources. Once a squirrel was caught inside a commercial trap (Havahart Squirrel & Chipmunk Live Trap), the trap was placed in a large plastic storage box and immediately transported to the Centre for Functional and Metabolic Mapping at the University of Western Ontario (5-min transport time). Upon completion of imaging, 3-5 ml of warmed NaCl was administered subcutaneously. A small ear punch was made in squirrels to mark the animal and avoid repeated study of the same squirrel. A blanket and supplemental heat were provided for recovery. After recovery, squirrels were transported in the plastic storage/induction box back to the capture location and released. One squirrel died during recovery. Its brain was removed, fixed by submersion in formalin, and subsequently imaged for ultra-high resolution structural MRI at the University of Pittsburgh Brain Institute.
Reporting on sex	Rats were all male, one female squirrel, 8 female marmosets. Availability of sex of each species depended on availability (with less marmosets due to females used for breeding; only squirrels who entered the trap were used).
Field-collected samples	This study did not involve field-collected samples.
Ethics oversight	Experimental procedures were in accordance with the Canadian Council of Animal Care policy and a protocol approved by the Animal Care Committee of the University of Western Ontario Council on Animal Care.

Note that full information on the approval of the study protocol must also be provided in the manuscript.

Magnetic resonance imaging

Experimental design

Design type	Resting-state fMRI.
Design specifications	Spontaneous fluctuations at rest.
Behavioral performance measures	NA. Physiology monitored for depth of anesthesia only.

Acquisition

Imaging type(s)	functional, structural
Field strength	9.4 T and 7 T
Sequence & imaging parameters	For both rats and grey squirrels, data were acquired on a 9.4 T 31 cm horizontal bore MRI scanner (Varian/Agilent, Yarnton, UK) and Bruker BioSpec Avance III console with the software package Paravision-6 (Bruker BioSpin Corp, Billerica, MA) and a custom-built, high-performance 15-cm-diameter gradient coil with 400-mT/m maximum gradient strength (41) at the Centre for Functional and Metabolic Mapping at the University of Western Ontario. The animal holders and radiofrequency receive arrays were built in-house, and design files for these stereotactic holders have been made available (25) with geometrically optimized phased array receive coil designs for the two species. The rat coil was made up of 6 channels, while a larger marmoset coil with 8 channels was used for the squirrels. Both coils have a similar signal-to-noise ratio (SNR). Preamplifiers were located behind the animals, and the receive coil was placed inside a quadrature birdcage coil (12-cm inner diameter) used for transmission.<br>For the rats, functional imaging was acquired during one session for each animal, with 6 functional runs (at 600 volumes each) with the following parameters: TR = 1,500 ms, TE = 15 ms, field of view = 38.4 ⍰ 38.4 mm, matrix size = 96 ⍰ 96, voxel size = 0.4 ⍰ 0.4 ⍰ 0.4 mm, slices = 35, bandwidth = 280 kHz, GRAPPA acceleration factor: 2 (anterior-posterior). T2-weighted structural scans were acquired for each animal with the following parameters: TR = 7,000 ms, TE = 44 ms, field of view = 38 ⍰ 38 mm, matrix size = 192 ⍰ 192, voxel size = 0.2 ⍰ 0.2 ⍰ 0.4 mm, slices = 35.<br>The squirrel imaging was similar to rat imaging with functional imaging data acquired from one session for each animal

with 3-4 functional runs (at 600 volumes each) with the following parameters: TR = 1,500 ms, TE = 15 ms, flip angle = 40 degrees, field of view = 64 □ 64 mm, matrix size = 128 □ 128, voxel size = 0.5 □ 0.5 □ 0.5 mm, slices = 42, bandwidth = 400 kHz, GRAPPA acceleration factor: 2 (anterior-posterior). T2-weighted structural scans were acquired for each animal with the following parameters: TR = 7,000 ms, TE = 55 ms, field of view = 64 □ 64 mm, matrix size = 384 x 384, voxel size = 0.133 □ 0.133□ 0.5 mm, slices = 45. As an in vivo registration template, we also acquired a structural T2-weighted image from squirrel #5 with an isotropic voxel resolution with the following parameters: TR = 15,000 ms, TE = 48 ms, field of view = 51 x 51 mm, matrix size = 256 x 256, voxel size = 0.2 □ 0.2 □ 0.2 mm, bandwidth = 50 kHz, slices = 100, number of averages = 3.
For the squirrels, an ultra-high-resolution ex vivo template was also generated from squirrel #5 – the same brain used as the in vivo registration template. The sample was fixed by submersion in 10 % formalin for one week, then imaged at the University of Pittsburgh Brain Institute on a 9.4 T 30 cm horizontal bore MRI scanner (Bruker BioSpin Corp, Billerica, MA) equipped with a Bruker BioSpec Avance Neo console and the software package Paravision-360 (version 3.2; Bruker BioSpin Corp, Billerica, MA) and a custom high performance 17 cm gradient coil (Resonance Research Inc, Billerica, MA) performing at 450 mT/m gradient strength. To maximize sensitivity, a custom 30 mm inner diameter millipede quadrature coil (ExendMR LLC, Milpitas, CA) was used. The sample was placed in a 50 ml conical centrifuge tube, filled with Fomblin oil (Solvay Solexis, West Deptford, NJ), and placed under vacuum (-27 inHg) for 30 minutes to remove air bubbles. A T2*-weighted structural scan was acquired with the following parameters: TR = 100 ms, TE = 8 ms, field of view = 35 x 30 x 20 mm, matrix size = 700 x 600 x 400, voxel size = 0.05 x 0.05 x 0.05 mm, bandwidth = 50 kHz, number of averages = 2, total scan time = 14 hours, 48 minutes. The T1 relaxation time of the tissue was measured (mean T1 = 1,047 ms), and an optimum flip angle (Ernst angle) of 24.7 degrees was set for a TR of 100 ms.
For the marmosets, functional imaging was acquired from two sites. The University of Western Ontario data were acquired using the same 9.4 T scanner as the rat and squirrel data. A custom 5-channel receive coil was used, which was rigidly fixed to the head implant (42). Radiofrequency transmission was accomplished with a quadrature birdcage coil (12-cm inner diameter) built in-house. Functional imaging was performed over multiple sessions (days) for each animal, with 4-6 functional runs (at 600 volumes each) per animal with the following parameters: TR = 1,500 ms, TE = 15 ms, flip angle = 35 degrees, field of view = 64 □ 64 mm, matrix size = 128 □ 128, voxel size = 0.5 □ 0.5 □ 0.5 mm, slices = 42, bandwidth = 500 kHz, GRAPPA acceleration factor: 2 (anterior-posterior). T2-weighted structural scans were acquired for each animal during one of the awake sessions with the following parameters: TR = 5,500 ms, TE = 53 ms, field of view = 51.2 □ 51.2 mm, matrix size = 384 □ 384, voxel size = 0.133 □ 0.133 □ 0.5 mm, slices = 42, bandwidth = 50 kHz, GRAPPA acceleration factor: 2.
The NIH marmoset data were acquired using at 7 T 30 cm horizontal bore magnet (Bruker BioSpin Corp, Billerica, MA, USA) with the software package Paravision (Bruker BioSpin Corp, Billerica, MA, USA), a 15-cm-diameter gradient coil with 450-mT/m maximum gradient strength (Resonance Research Corp, Billerica, MA, USA) and custom 10-channel phased-array receive coil which conformed to the 3D printed head holder. Radiofrequency transmission was accomplished with a 16-rung high-pass birdcage coil. Functional imaging was performed in a single session with 4-8 functional runs (at 512 volumes each) with the following parameters: TR = 2,000 ms, TE = 22.2 ms, flip angle = 70.4, field of view = 28 × 36 mm, matrix size = 56 × 72, voxel size = 0.5 □ 0.5 □ 0.5 mm, slices = 38. Two sets of spin-echo EPI with an opposite phase-encoding direction (left-right and right-left) were collected for the EPI-distortion correction (TR= 3,000 ms, TE = 0.44 ms, flip angle = 90 degrees, FOV= 28 × 36 mm, matrix size = 56 × 72, voxel size = 0.5 □ 0.5 □ 0.5 mm, axial slices = 38). T2-weighted structural image scans were acquired for each animal during one of the awake sessions with the following parameters: TR= 6,000 ms, TE = 9 ms, flip angle = 90°, FOV= 28 × 36 mm, matrix size = 112 × 144, slices = 38, voxel size = 0.25 × 0.25 × 0.5 mm, number of averages = 8.

Area of acquisition: Whole brain.

Diffusion MRI: ☐ Used ☒ Not used

Preprocessing

Preprocessing software:

FMRIB Software Library v6.0
AFNI Version AFNI_20.0.19 'Galba'
ANTs Version 5

For squirrels, rats, and marmosets, data were similarly processed with custom preprocessing pipelines using the Analysis of Functional NeuroImages (AFNI)(43) and FMRIB Software Library (FSL)(44) software packages. Raw functional images were converted to NifTI format using dcm2niix (45) and reoriented from the sphinx position using FSL. The images were then despiked (AFNI's 3dDespike), and volume registered to the middle volume (AFNI's 3dvolreg). The motion parameters from volume registration were stored for later use with nuisance regression. For the rats, images were smoothed by a 1 mm full-width at half-maximum Gaussian kernel to reduce noise (AFNI's 3dmerge); for the larger squirrel and marmoset brains, a 1.5 mm kernel was used. An average functional image was then calculated for each run and registered (FSL's FLIRT) to each animal's T2-weighted image – the 4D time-series data was transformed using this matrix. T2-weighted images were manually skull-stripped (including the olfactory bulb in all three species), and this mask was applied to the functional images.
Each individual animal's T2-weighted images were non-linearly registered to high-resolution anatomical templates: for squirrels, images were registered to the higher-resolution anatomical image from squirrel 5 (voxel size = 0.2 x 0.2 x 0.2 mm). For rats, images were registered to the anatomical image provided in template space (voxel size = 0.05 x 0.05 x 0.05 mm) (46). For marmosets, images were registered to the NIH marmoset brain atlas (voxel size = 0.2 x 0.2 x 0.2 mm) (47). Functional images from all three species were bandpass filtered between 0.01 and 0.1 Hz.

Normalization: FSL's FLIRT (12 parameter), then non-linear transformation using Advanced Normalization Tools

Normalization template: NIH Marmoset Brain Atlas (Version 3), Paxinos Rat Atlas

Noise and artifact removal: See above.

Volume censoring | Initial (stabilization) volumes were censored via the scanner console before raw images were saved.

Statistical modeling & inference

Model type and settings: multivariate

Effect(s) tested: Pearson correlation.

Specify type of analysis: ☐ Whole brain ☐ ROI-based ☒ Both

Anatomical location(s): Frontal seeds: posterior cingulate cortex (PCC), posterior parietal cortex (PPC), the primary somatosensory cortex (S1), the primary motor cortex (M1), insular cortex (Ins), striatum (Str), pulvinar (Pul), and superior colliculus (SC). Target region abbreviations: Frontal association cortex (FrA); lateral orbital cortex (LO); medial orbital cortex (MO); frontal cortex, area 3 (Fr3); secondary motor cortex (M2); frontal areas 1 – 6 (F1-F6); area 6 of cortex, ventral, part a (6VA); area 6 of cortex, dorsorostral part (6DR); area 8a or cortex, dorsal part (8AD); area 8a of cortex, ventral part (8AV); area 45 of cortex (45); area 47 of cortex (47); area 46 of cortex, dorsal part (46D); area 46 of cortex, ventral part (46V).

Statistic type for inference (See Eklund et al. 2016): NA

Correction: NA

Models & analysis

n/a	Involved in the study
☐	☒ Functional and/or effective connectivity
☒	☐ Graph analysis
☒	☐ Multivariate modeling or predictive analysis

Functional and/or effective connectivity: Pearson correlation.
